# Assessing the post-release effects of capture, handling and placement of satellite telemetry devices on narwhal *(Monodon monoceros)* movement behaviour

**DOI:** 10.1093/conphys/coaa128

**Published:** 2021-01-07

**Authors:** Courtney R Shuert, Marianne Marcoux, Nigel E Hussey, Cortney A Watt, Marie Auger-Méthé

**Affiliations:** 1 Department of Integrative Biology, University of Windsor, Windsor, ON N9B 3P4, Canada; 2 Arctic Aquatic Research Division, Fisheries and Oceans Canada, Winnipeg, MB R3T 2N6, Canada; 3 Department of Biological Sciences, University of Manitoba, Winnipeg, MB R3T 2N2, Canada; 4 Department of Statistics, University of British Columbia, Vancouver, BC V6T 1Z4, Canada; 5 Institute for the Oceans & Fisheries, University of British Columbia, Vancouver, BC V6T 1Z4, Canada

**Keywords:** Arctic, cetacean, accelerometry, behaviour, handling response

## Abstract

Animal-borne telemetry devices have become a popular and valuable means for studying the cryptic lives of cetaceans. Evaluating the effect of capture, handling and tagging procedures remains largely unassessed across species. Here, we examine the effect of capture, handling and tagging activities on an iconic Arctic cetacean, the narwhal (*Monodon monoceros*), which has previously been shown to exhibit an extreme response to extended capture and handling. Using accelerometry-derived metrics of behaviour, including activity level, energy expenditure and swimming activity, we quantify the post-release responses and time to recovery of 19 individuals following capture and tagging activities considering the intrinsic covariates of sex and individual size and the extrinsic covariates of handling time and presence of a ‘bolt-on’ satellite telemetry device. From accelerometer-derived behaviour, most narwhals appeared to return to mean baseline behaviour (recovery) within 24 hours after release, which was supported by longer-term measures of diving data. None of the covariates measured, however, had an effect on the time individuals took to recover following release. Using generalized additive models to describe changes in behaviour over time, we found handling time to be a significant predictor of activity levels, energy expenditure and swimming behaviour following release. Individuals held for the longest period (>40 min) were found to display the largest effect in behaviour immediately following release with respect to swimming behaviour and activity levels. We also found some support for relationships between activity levels, energy expenditure and swimming activity and two other covariates: sex and the attachment of a bolt-on configuration satellite tags. Our results indicate that narwhals recover relatively quickly following capture, handling and tagging procedures, but we suggest that researchers should minimize handling time and further investigation is needed on how to mitigate potential effects of bolt-on satellite tags in these sensitive species.

## Teaser

Narwhals, iconic Arctic cetaceans, were monitored after routine capture and tagging. Using accelerometry-derived behaviour, most individuals appeared to recover within hours post-release. Some evidence suggests that handling time, sex and the presence of bolt-on satellite tags may have had an effect on changes in behaviour, highlighting the sensitivity of this species.

## Introduction

Telemetry technology has advanced substantially over the past few decades resulting in sophisticated electronic tags or tag packages that are now smaller than ever (Evans *et al.*, 2013; Lennox *et al.*, 2017). Animal-borne telemetry devices are particularly useful for the study of aquatic animals for which direct observation is often prohibited by a cryptic lifestyle or for those that live in remote and largely inaccessible environments (Walker *et al.*, 2012; Hussey *et al.*, 2015). To date, telemetry devices have provided unprecedented insights into migration routes and timing (e.g. Hauser et al. 2014), diving behaviour (e.g. Owen *et al.*, 2019), three-dimensional movement (e.g. Fahlman *et al.*, 2008), swimming speed (e.g. Williams *et al.*, 2004) and physiology (e.g. Andrews and Enstipp, 2016). While these data are proving powerful in management and conservation contexts (Brooks *et al.*, 2017; Hays *et al.*, 2019), there is increasing awareness of animal welfare related to invasive tagging procedures that has naturally led to a call from regulatory agencies for the development of taxon-specific guidelines for their application (Gales *et al.*, 2009). Many refinement frameworks also highlight the need to assess considerations on a species-specific level (Hawkins, 2004) as well as across all aspects of experimental design for biologging studies (Casper, 2009). As a result, several expert groups pooled collective knowledge from field activities, as well as previous work assessing the use and placement of telemetry devices, to create best practice recommendations for pinnipeds (Horning *et al.*, 2017b, 2019) and cetaceans (Andrews *et al.*, 2019). These best-practice recommendations provide an important framework moving forward and emphasize the need for data to examine how animal handling and the placement of telemetry devices potentially impact health, behaviour and survival of marine mammals.

Cetaceans, especially, have seen a rapid increase in the number of individuals fitted with telemetry devices over recent years (McIntyre, 2014; Andrews *et al.*, 2019). These telemetry devices are generally attached using either a non-invasive method, such as suction cup mounting (Goldbogen *et al.*, 2013), or through more invasive anchored mounts along the dorsal fin or ridge, such as the ‘limpet’-style tag (Andrews *et al.*, 2015), and can often be attached without directly restraining individuals by using a pole or cross-bow to achieve short-term (days) and medium-term (weeks) deployment durations. Attachment techniques for long-term deployments (months) using ‘bolt-on’ configurations, however, often require capture and handling (Balmer *et al.*, 2011; Heide-Jørgensen *et al.*, 2017). While researchers follow best practices for capture, handling and tag attachment, approved through Animal Care Committees in respective organizations, assessments of the impact of these procedures on cetaceans are limited (Andrews et al. 2019).

Narwhals (*Monodon monoceros*) are an iconic medium-sized odontocete and a culturally important species ranging across much of the Eastern Arctic. Narwhals exhibit sexual dimorphism, both in terms of body size and most notably, by the presence of a tusk that primarily occurs in males (Reeves and Tracey, 1980; Petersen *et al.*, 2012; Heide-Jørgensen, 2018). In the Canadian Arctic, narwhals typically spend the largely ice-free summer months in and around fjords, inlets and sounds (Heide-Jørgensen *et al.*, 2013). Using animal-borne telemetry devices, previous work has documented their yearly life cycle and movement patterns (Heide-Jørgensen *et al.*, 2002, 2006; Laidre and Heide-Jørgensen, 2005), deep-diving capabilities (Laidre and Heide-Jørgensen, 2005; Heide-Jørgensen *et al.*, 2015; Watt *et al.*, 2015), responses to predators (Laidre *et al.*, 2006; Breed *et al.*, 2017), novel behaviours such as upside-down swimming at depth (Dietz *et al.*, 2007) and potential for extreme sensitivity to future climate scenarios (Williams *et al.*, 2011). However, studies have also revealed disturbance associated with extended animal handling during the capture and tag attachment process (Williams *et al.*, 2011, 2017a). The response of individuals to handling periods greater than 60 minutes was characterized by extreme bradycardia coupled with higher swimming effort and deep diving (Williams *et al.*, 2017a). Such a strong response by narwhals post-tagging is concerning and highlights the need for a greater understanding of the effects of capture, handling and tagging beyond the first 90 minutes post-release that were the focus of Williams *et al.* (2017a).

Animal-borne accelerometers provide a tool to quantify behavioural effects following capture and tagging events through recording high-resolution, continuous three-dimensional movement data over time. Several metrics of behaviour have been extracted from accelerometry data across a wide variety of species, including changes in activity levels (norm of jerk, defined as the square-root of the sum of squares for differential of acceleration in all axes; Ydesen *et al.* 2014; Barkley et al. 2020), approximate energy expenditure (Vectorial Dynamic Body Acceleration, VeDBA; Broell *et al.*, 2016; Stothart *et al.*, 2016; Udyawer *et al.*, 2017) and, most often for aquatic animals including narwhal, swimming behaviour (tail-beat frequency or stroke rate; Williams *et al.* 2004, 2017a, b; Sato *et al.*, 2007; Martin Lopez *et al.*, 2015; Ladds *et al.*, 2017). Diving response has also been shown to be a key indicator of how individuals and populations respond to disturbance (DeRuiter *et al.*, 2013; Miller *et al.*, 2015; Williams *et al.*, 2015; van Beest *et al.*, 2018; Warren *et al.*, 2020). Quantifying the degree of departure of animal behaviour from the normal or baseline values following capture and tagging events and the length over which detrimental effects modify behaviour remains an important part of holistically evaluating research activities (Wilson and McMahon 2006). Through the use of data from individuals equipped with biologging devices, it is possible to assess across- and within-individual changes in behaviour following capture and tagging using the above metrics. Specifically, a return to a long-term mean in a given behavioural index (i.e. baseline behaviour), provides an indicator of recovery for that metric following invasive research activities.

We used a combination of behavioural metrics derived from accelerometers coupled with dive data to characterize both the post-release behaviour and the time to return to baseline behaviour of narwhals following capture and tagging events across three independent field programs. Specifically, we aimed to characterize activity levels, energy expenditure and swimming behaviour across- and within-individuals following release from capture and tagging events to assess the effect of handling time on these behavioural metrics for the first 72 hours post-release (defined by the accelerometer sampling duration). Furthermore, we examined time to recovery to baseline behaviour (measured here as the return of individual deviance of baseline behaviour to zero, baseline measured beyond 36 hours after release) and assessed if key covariates (sex, handling time and the presence of a bolt-on tag) may have influenced behaviour and recovery times. By using longer-term measures of dive behaviour (baseline measured 7–14 days post-release), we further aimed to determine if the relatively short behavioural window provided by acceleration data accurately captured the period of recovery in narwhals.

## Methods

### Study site and capture, handling and tagging protocols

Over three summer seasons in 2012, 2017 and 2018, a total of 29 narwhals were captured and equipped with satellite transmitting tags and/or recoverable biologging devices in Tremblay Sound (72°21.389 N, −81°05.855 W) on northern Baffin Island, Nunavut, Canada (see Table 1). Tagging efforts were part of a larger program to monitor ecosystem-wide health in the high Arctic led by Fisheries and Oceans Canada’s long-term marine mammal monitoring program.

**Table 1:** Summary of study animals, including animal sex, animal length (cm), age class (adults classified as body length, >300 cm; Hay 1984), minutes held follow capture and prior to release (*t_cap_*) and the presence of a satellite tag attached via ‘bolt-on’ configuration. The type of accelerometer (ACC-type) is also included. The number of hours of acceleration data recorded while attached to each narwhal is also included (hours); however, several tags were programmed with delays in recording for a period of up to 4 hours (indicated by the ^*^)

Animal	Sex	Length (cm)	Age class	*t_cap_* (min)	Bolt-on tag?	Hours	ACC type
12–03	F	390	Adult	30	Y	5	TDR-DD
12–05	F	262	Juvenile	30	Y	15	TDR-DD
17–03	F	400	Adult	36	Y	182^*^	MBL
17–04	M	432	Adult	47	Y	38^*^	MBL
17–05	M	488	Adult	67	Y	48^*^	MBL
17–08	F	375	Adult	37	Y	83	Acousonde
17–09	F	385	Adult	36	Y	70^*^	Acousonde
17–10	M	400	Adult	30	N	189^*^	MBL
17–11	F	390	Adult	39	Y	12^*^	Acousonde
17–12	F	425	Adult	25	Y	15^*^	Acousonde
17–13	M	298	Juvenile	36	Y	26^*^	Acousonde
17–14	M	230	Juvenile	18	N	3^*^	Acousonde
17–18	F	370	Adult	31	Y	11	Acousonde
17–19	F	380	Adult	35	Y	15	Acousonde
17–20	F	408	Adult	35	Y	22	Acousonde
18–02	F	357	Adult	34	Y	112^*^	Acousonde
18–03	M	303	Adult	35	N	19^*^	Acousonde
18–04	F	382	Adult	23	N	107^*^	Acousonde

Narwhal were captured following standard protocols and monitored by a veterinarian (Orr *et al.*, 2001; Heide-Jørgensen *et al.*, 2015). In brief, a 50 m x 10 m gill net was set perpendicular to shore and monitored by a minimum of two people for the entire period set. Immediately following a narwhal entering the net, two boats were deployed: the first to locate the animals and pull the net to the surface to allow the individual/s to breathe and the second to release the net from the anchor buoy. Following release, a shore based team pulled the net to the beach, securing individual narwhal in approximately 50 cm of water with animals facing out to deeper water. A padded rope loop was applied around the tail stalk and the animal was disentangled from the net. Captured narwhal were then held by a minimum of four experienced handlers and equipped with satellite transmitting tags (various models including TDR10, Wildlife Computers, Inc. and CTD Oceanography SRDL with GPS, SMRU Instrumentation used to derive dive behaviour, see below) via spider wires crimped to three sub-dermal 10 mm pins made of Tecaform™ sterilized in Ethylene Oxide gas. An 11-mm stainless steel tube sharpened at one end was used to cut a path for insertion of each pin into the dorsal ridge. Custom-made lock washers were then placed on the pin to the point of skin contact with no pressure. Washers were locked in place and pins trimmed flush to the outer-side of the washer. Stainless steel cables attached to the tag were inserted through the washers, adjusted to the desired length and crimped to secure the tag to the animal (Orr *et al.*, 2001). All capture and tagging protocols were approved by the Fisheries and Oceans Animal Care Committee and a License for Scientific Purposes was granted.

**Figure 1:**
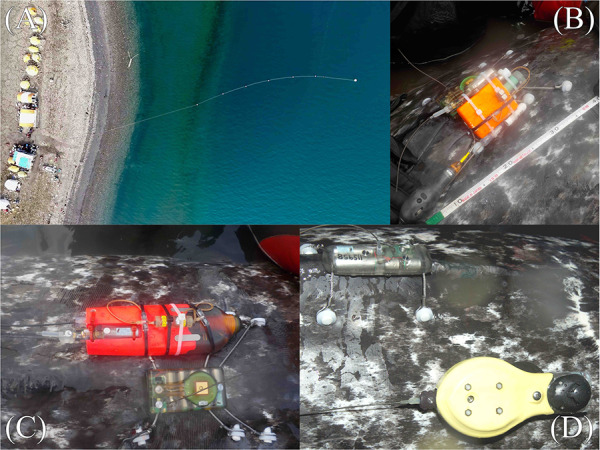
Images showing net deployment anchored perpendicular to shore (A) in Tremblay Sound (72°21.389 N, −81°05.855 W), an example of attachment of the bolt-on configuration (3 pins) for a TDR-10 satellite tag, and accompanying (B) Maritime Biologgers (MBL) deployment package with timed release mechanism attached to pins or (C) Acousonde unit attached via both a suction cup mounting and little Leonardo releases held across the spider wires holding the TDR-10 satellite tag to the dorsal ridge and (D) a Daily Diary (TDR-DD) tag attached with a suction cup under a bolt-on satellite tag (2 pins).

Of the 29 narwhal captured over the three field seasons, 20 were outfitted with a telemetry device that included an accelerometer unit; either an Acousonde™ (*n* = 13; Model B003B, Greeneridge Sciences, Inc.), a daily diary (*n* = 3; TDR-DD, Wildlife Computers, Inc.) or a Maritime BioLoggers (MBL) accelerometer (*n* = 4). Accelerometers (Acousounde, TDR-DD and MBL) were fixed within recoverable biologging packages that consisted of a float for recovery (i.e. to bring the device to the surface) and a SPOT and VHF tag to locate the unit once at the surface. Accelerometer packages were attached to the narwhal posterior to the dorsal ridge via a suction cup and/or tethered line attached to one pin of the main satellite tag via release timers (Little Leonardo; see above, Fig. 1, Table 1). Accelerometers were programmed to remain attached on animals for 3 to 7 days (mean deployment duration 53.9 hours). The Acousonde units primarily sampled high-resolution acoustic data, but were also programmed to sample tri-axial magnetometry and acceleration as well as other auxiliary data channels including light level, pressure and temperature (Burgess, 2009). Sampling rate was set at 800 Hz, but for the purposes of this study was subsampled at 50 Hz (± 4 *g*). One individual Acousonde unit, however, failed to collect acceleration data and was not considered further in the analysis. MBL units were programmed to sample acceleration at 50 Hz with a 4 *g* range. The TDR10-Daily Diary tag was housed in a float pack with a SPOT5 tag from Wildlife Computers. Each float pack unit was suction cupped onto the side of the narwhal dorsal ridge and were not tethered to the satellite tag. The SPOT5 tag provided location information useful for relocating the tag after release from the animal, while the Daily Diary tags sampled high-resolution acceleration, orientation and speed (via paddle wheel), through the use of an accelerometer and magnetometer, and also include measurements of depth, temperature and light (Wilson *et al.*, 2008). Daily Diary tags sampled acceleration at 16 Hz ± 2 *g*. For one individual equipped with a TDR-DD, the tag fall off within one hour of deployment and consequently it was not included in analysis.

### Post-release behaviour assessed using accelerometer-derived metrics

**Figure 2:**
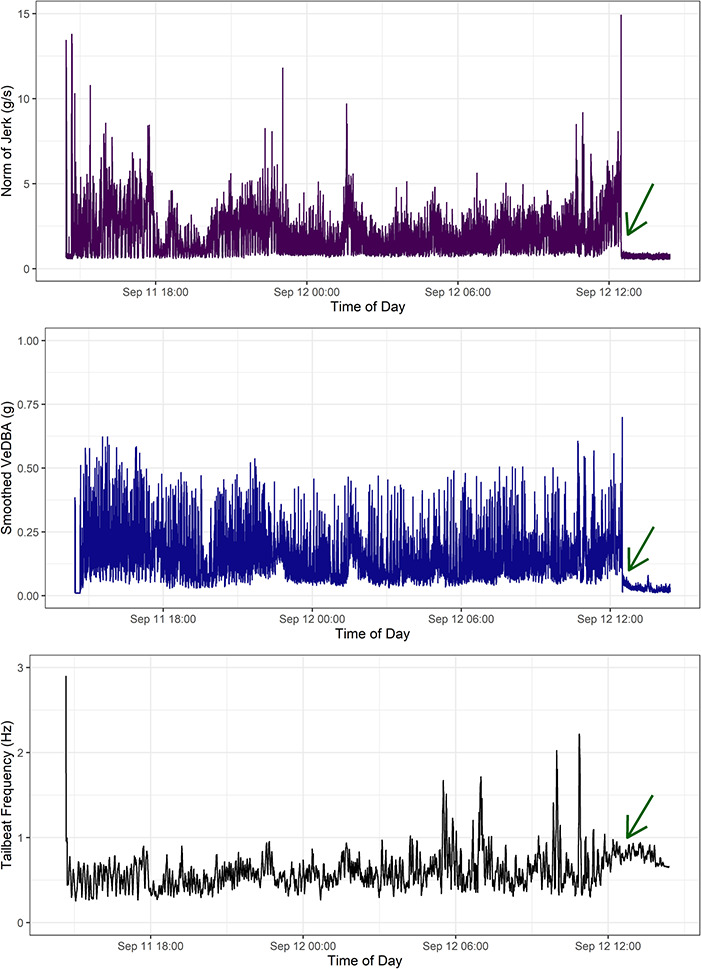
Example of behaviour across the full recording period (~ 22 hours) for a 3.2 m male narwhal 17–20, including the period immediately after release. Behavioural metrics include (a) activity levels (norm of jerk), (b) energy expenditure (sVeDBA) and (c) swimming activity (TBFreq), before the tag fell off just after 12:00 on the second day as indicated by the green arrows.

We used three behavioural metrics of energy expenditure, swimming behaviour and activity levels from accelerometer data to examine post-release behaviour. First, we used a smoothed vector of Vectorial Dynamic Body Acceleration (sVeDBA) as a proxy for coarse energy expenditure (Miwa *et al.*, 2015; Broell *et al.*, 2016; Udyawer *et al.*, 2017; Grémillet *et al.*, 2018; Wilson *et al.*, 2019). To derive static acceleration (relative animal body position and postural dynamics with respect to gravity), raw acceleration data for each deployment were first low-pass filtered in each axis at 0.1 Hz (Shepard *et al.*, 2008). Dynamic acceleration was then derived by subtracting static acceleration from raw acceleration data in each axis (Shepard *et al.*, 2008) and VeDBA estimated by taking the vectorial sum of dynamic acceleration across all three orthogonal axes and smoothing using a 3 s moving window (Fig. 2; Stothart *et al*. 2016). Second, to quantify activity level, the derivative of acceleration, jerk, was calculated by taking the differential of raw acceleration in each axis and then computing the norm of jerk (Fig. 2; Ydesen *et al.* 2014). Third, we estimated tail-beat frequency per second as a measure of swimming behaviour. Tail-beat frequency (TBFreq) was derived through fast Fourier transform of dynamic acceleration in the Z-axis, approximating dorso-ventral movement, within a 9-second moving window around each second to minimize spectral leakage. The primary frequency was extracted at each second (Fehlmann *et al.*, 2017). Derived TBFreq was then smoothed using a running mean of 60 seconds to remove flow noise associated with swimming or wiggle from the tag attachment (Cade *et al.*, 2017); any spikes in frequency above 1 Hz remaining are likely due to artefacts from a quick movement experienced by the tag or where suction cups may have been dislodged (Fig. 2).

We were interested in how these three behavioural metrics estimated for each narwhal deviated from long-term mean values following release from capture, handling and tagging activities. For modelling purposes, each accelerometer-derived behavioural metric was summarized as an hourly mean value. To calculate within-individual deviance for each metric, we calculated a pooled mean value using data beyond the first 36 hours to act as a baseline. Pooled mean values were separated by tag types to account for differences in attachment set up (Acousonde and MBL/TDR-DD; see Table 1). These pooled means were then subtracted from hourly mean values for each individual/tag type in order to calculate within-individual deviance for each behavioural metric. While any pooled mean value for ‘baseline behaviour’ would ideally be derived from data several days post-release for each individual separately (e.g. Dechen Quinn *et al.* 2012), we were limited in sampling duration for many of the accelerometers because most units detached from the narwhals earlier than expected (e.g. only 6 individuals had usable acceleration data > 48 hours post-release; Table 1). As a result of this limitation, we explored variation in the three behavioural metrics for the first 72 hours post-release. We are confident that this represents an acceptable time frame for assessment given that several studies have noted that post-escape or post-disturbance behaviour returns to normal within the first few hours up to one day across a variety of species (Gales *et al.*, 2012; Hastie *et al.*, 2015; Russell *et al.*, 2016; Whitney *et al.*, 2016; Williams *et al.*, 2017a; Blackwell *et al.*, 2018; van Beest *et al.*, 2018), and our longer-term diving data indicated that narwhals recovered within 36 hours (see below).

### Modelling recovery

#### Time to recovery

We determined the time point at which both the population of narwhal (across-individuals) and individual animals (within-individual) had recovered, or returned to baseline behaviour. Across-individuals, patterns in mean hourly deviance for each of the three behavioural metrics were plotted for the first 72 hours post-release. The time at which mean hourly deviance was no longer significantly different from zero was used as a measure of when the population of individuals had recovered, i.e. behavioural metrics returned to expected values of ‘normal’ behaviour (Dechen Quinn *et al.*, 2012; Thiemann *et al.*, 2013; Rode *et al.*, 2014). Within-individuals, the mean time (hour) in which centred-values were no longer significantly different from zero across all three behavioural metrics (within 95% confidence interval for within-individual deviance) was used to define the point at which individuals recovered to baseline values or assumed normal behaviour (Dechen Quinn *et al.*, 2012; Thiemann *et al.*, 2013; Rode *et al.*, 2014). Individual recovery points to baseline values were compared among individuals with additional covariates using generalized linear models with a Poisson distribution and log-link and were ranked based on evidence (Zuur *et al.*, 2009). Additional individual covariates comprised (i) with or without ‘bolt-on’ configuration of satellite telemetry device, (ii) sex, (iii) age class (adult vs. juvenile delineated based on standard length of individuals >300 cm; Hay, 1984) and (iv) handling time (categorical; short: *t_cap_* < 30 min; medium: 30–40 min; long: > 40 min).

#### Behavioural response following capture, handling and tagging

To understand individual narwhal post-release behaviour relative to tagging procedures and intrinsic factors (sex and size), we examined the temporal changes in individual deviance for each of the accelerometer-derived metrics with a set of generalized additive models. Given we were interested in how behaviours are modified post-release, our base models described changes in all three metrics as a function of time since release (*t_rel_*). Relationships were not linear, consequently single thin-plate smoothing splines were applied, with an additional shrinkage penalty in order to obtain the simplest spline and allow overly complex relationships to shrink to zero (Wood 2006). Models were designed *a priori* for relationships of interest as well as to deal with collinearity across model covariates (including satellite tag presence, sex, age class and handling time described above) and relatively small sample size. Each of the categorical covariates was modelled as either parametric covariates or as varying-coefficient smoothing interactions separately. All generalized additive modelling was performed within the R package ‘mgcv’ (Wood, 2006). All candidate models were ranked based on Akaike Information Criterion, corrected for small sample size (AICc; Burnham and Anderson, 2002). All analyses were conducted using R v.3.6.3 (R Core Team 2020).

### Post-release dive behaviour to validate accelerometry recovery estimates

To complement the accelerometer-based time to recovery analysis, we investigated diving data collected on a subset of the narwhals (*n* = 10). These time series depth data were collected over a considerably longer period than the accelerometer data and thus provides a means to determine if the 72 hour time frame used for the three accelerometer-derived behavioural metrics was adequate. While changes in diving behaviour are a useful indicator to investigate post-disturbance events (DeRuiter *et al.*, 2013; Miller *et al.*, 2015; Williams *et al.*, 2015; van Beest *et al.*, 2018; Warren *et al.*, 2020), the small sample size of useable time series dive data limited our capacity to investigate individual differences in dive behaviour through additive modelling. Diving behaviour were derived from satellite telemetry devices (TDR10 and CTD-SRDL; recorded approximately every 75 s) and quantified by taking hourly estimates of mean and maximum dive depths (meters). To determine the approximate normal dive behaviour of an individual, we calculated a long-term average of mean and max depths by averaging all data between 7 and 14 days following release. To determine deviance in dive behaviour across the first 72 hours, the long-term mean value was then subtracted from both hourly mean and maximum dive depths. Identical to our approach for analysing the three accelerometer metrics, we then used the time when the across-individual deviance for mean and maximum dive depth were not significantly different from 0 as the population-level time of recovery (Dechen Quinn *et al.*, 2012).

## Results

Acceleration data were recorded for an average of 53.9 hours across all narwhals sampled (*n* = 18; range, 3–189 hours; see Table 1); data, however, were not available for most individuals for the first four hours post-tag attachment/release due to programmed delays for auxiliary sampling (Table 1).

While mean hourly deviance for pooled data was not significantly different from zero for any of the three accelerometer-derived behavioural metrics over the first 72 hour period (Fig. 4A–C), narwhal overall were observed to be slightly more active and expend more energy for the first hour (Fig. 4A) and tail-beat frequency was slightly faster than normal for the first 24 hours (Fig. 4C). Wide confidence intervals further highlight high variability in accelerometry-derived behaviour following release. Individual recovery time points were generally reached within hours following release. At the individual level, mean estimated recovery time was 9 hours pooled across the three accelerometer-derived behaviours (range, 1–28 hours; excluding two individuals that had < 5 hours of accelerometer data). There was no significant difference in mean recovery time between male and female narwhal (β_sex_ = 0.21 ± 0.18, *z* = 1.15, *P* = 0.24), nor with respect to presence of a bolt-on satellite tag (β_tag_ = −0.10 ± 0.21, *z* = −0.51, *P* = 0.61). Binned handling time (*t_cap_*) as a predictor mean recovery time across individuals indicated that those held for medium durations (30–40 min) occasionally took longer to recover than those held for shorter durations (<30 min; β_medium_ = 0.32 ± 0.19, *z* = 1.66, *P* = 0.09), though these responses were highly variable and not significant. Individuals held for longer periods (>40 min) had a higher mean recover time value, however, these were not significantly different from those held for a short duration (β_long_ = 0.10 ± 0.30, *z* = 0.35, *P* = 0.72; see Fig. 5). However, all covariates were equal in explanatory power to that of a null model (< 3 ΔAICc; see Table S2).

Dive data indicated that narwhals spent significantly more time at shallower than average mean and maximum dive depths for the first 24 hours post-release (Fig. 4D, E). After this point, across-individual deviance in both dive metrics and their corresponding 95% confidence intervals were not significantly different from the long-term mean (0, based on data from 7–14 days post-release). This lends confidence that our accelerometer-based analysis was able to capture the post-release recovery period within the 72-hour time frame used.

**Figure 3:**
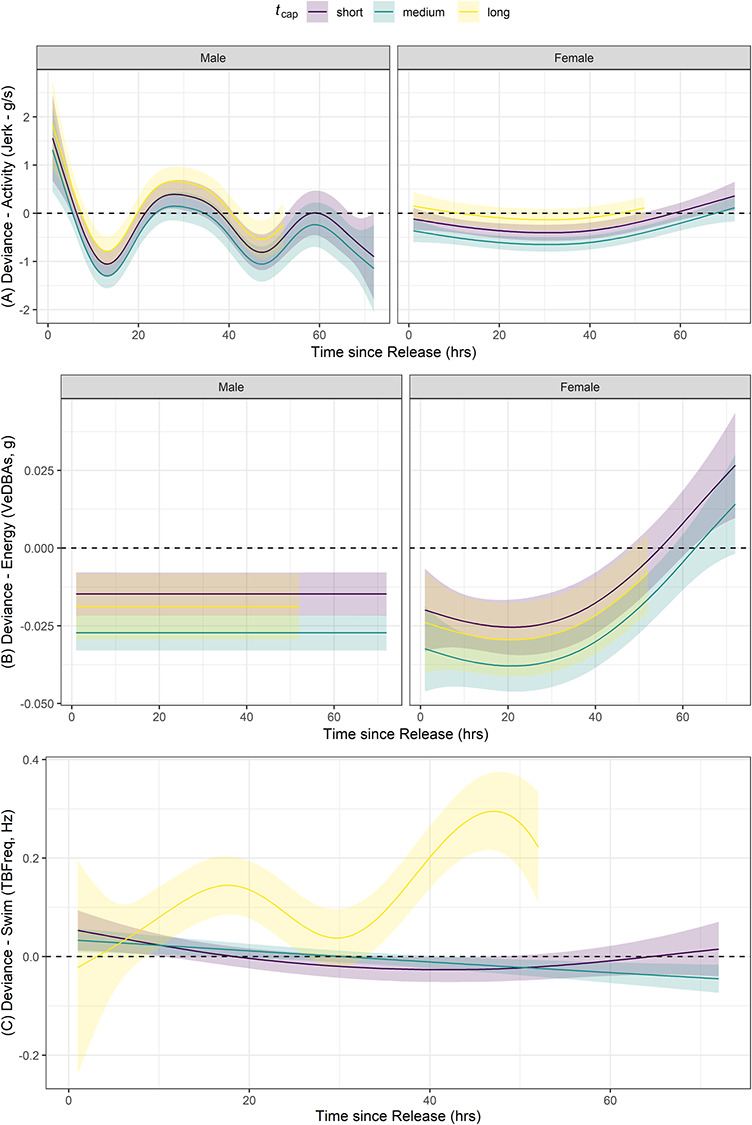
Plots of the interaction between time after release and sex as well as the additive effects relative to handling time, *t_cap_*, for deviance associated with (A) activity levels and (B) energy expenditure. Changes in swimming behaviour (C) were best explained by the interaction of time after release and handling time. Modelled behavioural responses relative to the population mean (deviance = 0) is highlighted by the horizontal dashed line. Data for individuals that were held for the longest time (*t_cap_* > 40 min) were only modelled to 50 hours as a result of limited accelerometry data. The spline calculated for male narwhal (B) was zeroed out as a result of the shrinkage penalty implemented to prevent overly complex relationships.

Deviance in narwhal activity level (jerk) was best explained by a thin-plate smooth with shrinkage of *t_rel_* that varied by sex and included the parametric effect of binned *t_cap_*, explaining 19.4% of model deviance (Table 2). Individuals who were held for longer periods (>40 min) tended to be more active in the first 72 hours following release (β_Long_ = 0.26 ± 0.11, *t* = 2.30, *P* = 0.02) than those held for short (<30 min) and medium durations (30–40 min; Fig. 3A), though the overall effect size was small (see Table S1). Energy expenditure (deviance - VeDBAs) was best explained by spline function of *t_rel_* that varied by sex as well as with the additional parametric effect of *t_cap_* (5.86 and 7.02% model deviance explained, respectively; Table 2), but were roughly equivalent in model evidence (ΔAICc < 4). Females displayed the greatest change in energy expenditure following release, while males were modelled to maintain constant energy expenditure as a function of *t_rel_* after shrinkage penalization, indicating that males may expend less energy overall as compared to the population mean (Fig. 3B). The additional effects of *t_cap_* on energy expenditure, while significant, were extremely small (effect size ranging −0.01 to −0.004 for each *t_cap_* bin; Fig. 3B and Table S1). Swimming behaviour (deviance—TBFreq), however, was best explained by a spline function of *t_rel_* that varied by *t_cap_*, explaining 18.1% of model deviance (Table 2). Individuals held for the longest period of time (>40 min) displayed a higher than normal swimming activity over time (Fig. 3C). There was also evidence to suggest that sex or the presences of a ‘bolt-on’ satellite tag may have influenced swimming behaviour in the first 72 hours after release (both explaining 19.1% deviance, Table 2). Females and those with ‘bolt-on’ satellite tags were found to have an overall lower TBFreq, though the overall effect size was extremely small (β_sex_ = −0.04 ± 0.01, *t* = −3.19, *P* = 0.001; β_tag_ = −0.03 ± 0.01, *t* = −2.55, *P* = 0.01; Table S1). The relatively low percentage of across-individual deviance explained by all models suggests a high degree of variability in hourly behaviour data from these accelerometers. Age class was ultimately removed from analyses as a covariate as all but one juvenile individual (of *n* = 3) had data spanning more than 15 hours post-release. Full model output, including significance of the smoothing terms, can be found in Table S1.

**Table 2:** Model results for time-varying patterns in hourly post-release behaviour of narwhal following capture and handling. Time since release (*t_rel_*) was modelled as a thin-plate smoothing spline with shrinkage. Covariates, including animal sex and the presence of a bolt-on satellite tag (Sat.tag), and time held in captivity (binned *t_cap_*) were modelled as both parametric terms and as varying coefficient models (‘by =’). Models including age class were removed from model selection due to insufficient sample size. Best models are highlighted in bold, ranked based upon Akaike Information Criterion (AICc), corrected for small sample size and deviance explained

	Activity			Energy expenditure			Swimming		
Model parameters	AICc	Δ AICc	Deviance explained	AICc	Δ AICc	Deviance explained	AICc	Δ AICc	Deviance explained
null s(*t_rel_*)	1632.18	81.99	5.9%	−1894.79	15.21	3.9%	−647.54	95.9	1.7%
Sex + s(*t_rel_*)	1609.78	59.59	9.6%	−1894.57	15.43	4.3%	−689.54	53.9	8.7%
Sat.Tag + s(*t_rel_*)	1629.2	79.01	6.7%	−1889.57	20.43	3.3%	−645.81	97.63	1.8%
*t_cap_* + s(*t_rel_*)	1604.3	54.11	10.7%	−1898.71	11.29	5.2%	−706.66	36.78	11.6%
s(*t_rel_*, by = Sex)	1571.95	21.76	15.9%	**−1906.44**	**3.56**	**5.8%**	−649.37	94.07	2.3%
s(*t_rel_*, by = Sat.Tag)	1651.58	101.39	1.1%	−1891.32	18.68	3.2%	−652.88	90.56	3.5%
*t_cap_* + s(*t_rel_*, by = Sex)	**1550.19**	**0**	**19.4%**	**−1910.00**	**0**	**7.0%**	−706.14	37.3	11.7%
*t_cap_* + s(*t_rel_*, by = Sat.Tag)	1620.61	70.42	8.4%	−1892.22	17.78	4.0%	−714.95	28.49	13.5%
s(*t_rel_*, by = *t_cap_*)	1569.35	19.16	17.9%	−1894.65	15.35	4.2%	**−737.22**	**6.22**	**18.1%**
Sex + s(*t_rel_*, by = *t_cap_*)	1565.63	15.44	18.7%	−1893.58	16.42	4.4%	**−743.44**	**0**	**19.1%**
Sat.Tag + s(*t_rel_*, by = *t_cap_*)	1563.66	13.47	19.0%	−1889.57	20.43	13.4%	**−742.29**	**1.15**	**19.1%**

## Discussion

Evaluating the behavioural responses of individuals following tagging and capture events remains an important goal for maintaining and evaluating best practices for telemetry studies in cetaceans. Generally, studies of tagging effects have focused on wound healing assessed by photographic resighting of tagged individuals (Robbins *et al.*, 2013; McIntyre, 2014; Andrews *et al.*, 2015; Best *et al.*, 2015; Gendron *et al.*, 2015; Heide-Jørgensen *et al.*, 2017; Norman *et al.*, 2018) or improving issues of drag and cost of transport associated with carrying telemetry devices (van der Hoop *et al.*, 2018; Kyte *et al.*, 2019). Here, we used high-resolution accelerometry data to examine the behaviour of individual narwhal following routine capture and telemetry device application by way of evidence-based model ranking with covariates for individual characteristics, handling time and the application of ‘bolt-on’ telemetry devices.

**Figure 4:**
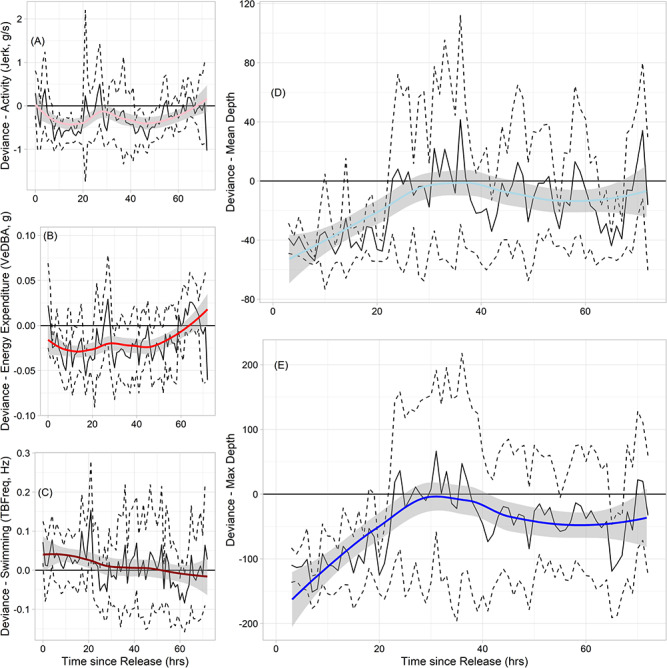
Mean population or across-individual hourly deviance with 95% confidence intervals (solid black and dashed lines, respectively) for accelerometer-derived behavioural metrics (A) activity levels, (B) energy expenditure, (C) swimming activity and (D, E) dive behaviour for the first 72 hours following release from capture/tagging events. Deviance was calculated by pooling hourly mean values for accelerometry data (36+ hours post-release, limited by measurement durations) and dive data (7–14 day hourly mean). Coloured lines represent loess-smoothed trend lines to further highlight trends in behaviour.

**Figure 5:**
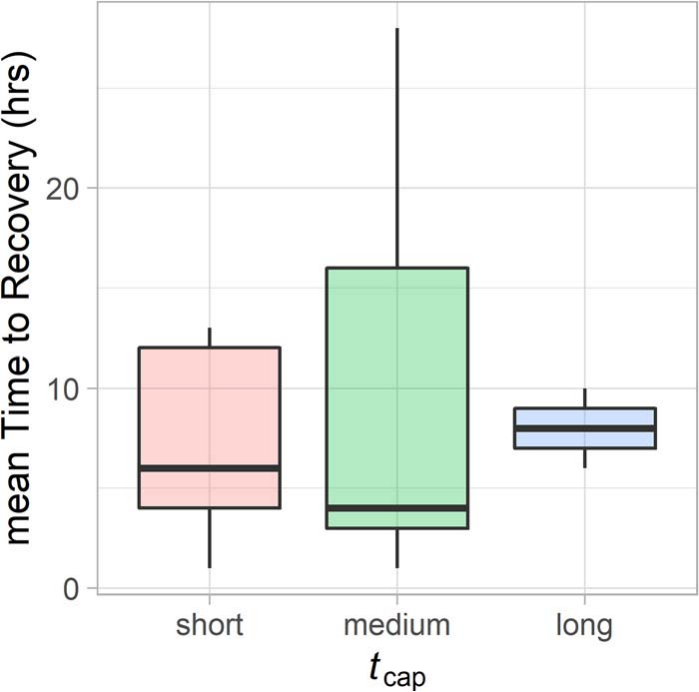
Summary of mean recovery time points (hours) after release as a function of handling time (*t_cap_*: short, <30 min; medium, 30–40 min; long, >40 min). Thick black bars in the middle of the boxes indicate the median value (bounded by the interquartile range) for time to recovery for each of the handling durations.

Generally, we found that individual narwhal recovered quickly from capture and tagging activities. While there was significant inter-individual variability in the magnitude of the response, most individuals appeared to recover in the first 8 hours following release with respect to accelerometry-derived behavioural metrics of activity levels, energy expenditure, and swimming behaviour. We also found no evidence to suggest that handling time had a major impact on recovery time. Other studies linking changes in behaviour have found individuals recover quickly following pile-driving activities (Russell *et al.*, 2016), mass-stranding events (Gales *et al.*, 2012) and with respect to tagging events (Whitney *et al.*, 2016; Warren *et al.*, 2020). Longer-term measures of diving behaviour in a subset of these narwhal further supported that most individuals returned to normal diving patterns within the first 24 hours after release. A study on harbour porpoise (*Phocoena phocoena*) noted that individuals exhibited shallower diving following both an initial handling event as well as a measured noise dose-response experiment, but also showed that the same individuals appear to have recovered to baseline behaviour within 24 hours or less (van Beest *et al.*, 2018). Similar shallow diving has been noted in narwhal in response to predation events and the presence of predators (Laidre *et al.*, 2006; Breed *et al.*, 2017), though these events resulted in longer disruptions of behaviour than measured here. Acoustically-tagged narwhal exhibited a period of silence (no vocalizations) after tagging in East Greenland, ranging from 9 to 37 hours after release (Blackwell *et al.*, 2018). The authors acknowledged, however, that this may be linked to narwhal being outside the feeding grounds as there was no relation to handling time with respect to the time to recovery (Blackwell *et al.*, 2018). Sperm whale (*Physeter macrocephalus*) tagged with suction cup telemetry devices via pole deployments also displayed lower clicking and buzzing rates, coupled with shallower dives, in the hours following tag deployment (Warren *et al.*, 2020).

Handling time was, however, found to be a significant covariate for the observed deviance in post-release behaviour for narwhals in the current study. Most individuals were held for an average of 35 minutes, which is at the lower end of handling times reported in previous work (Williams *et al.*, 2017a). Our models suggest that individuals held for longer periods of time tended to have higher than normal swimming activity immediately following release, as well as higher activity levels, but with little change in energy expenditure. This suggests that the few individuals held for greater than 40 minutes may have exhibited a greater escape response to handling events than those held for less time (Fig. 3). Previous work has noted that narwhals can exhibit extreme responses to handling events, sometimes expressing extreme bradycardia events and escape responses in the first 60 minutes following release (Williams *et al.*, 2017a). While Williams *et al.* (2017a) did not explicitly define a recovery period following tagging events, they considered a ‘post-escape period’ as occurring 45 to 90 minutes after release and found that individuals who were held for more than 60 minutes tended to display heart rate and stroke rate relationships more consistent with an escape response and cardiac freeze; those that were held for less than 60 minutes appeared to more quickly return to normal flight behaviours and cardiac output (Williams *et al.*, 2017a). Our accelerometer-derived behaviours appear to support this. We found that individuals held for the longest period displayed the largest change in activity and swimming behaviour following release, indicating that holding individuals for greater than 40 minutes resulted in a significant difference in post-release behaviours. Our diving behaviour proxies also appear to support the work of Williams *et al.* (2017a); narwhals here, all held for less than 60 minutes, generally appeared to dive to and spend the majority of their time at shallower than normal depths for the first 24 hours following release (Fig. 4), suggesting that individuals may have exhibited a temporary flight response. Similar dispersal to shallow water was noted in narwhal in the days following an attack by killer whales (*Orcinus orca*; Laidre et al. 2006). Even when individuals are not handled, cumulative exposure to research activities may still drive a similar dive response (Warren *et al.*, 2020).

In addition to handling time, this study also presents some evidence to suggest that individuals with ‘bolt-on’ satellite tags had a lower tail-beat frequency following release than those without. Several studies have been attempted to determine the effects of equipping animals with invasive telemetry devices in a wide variety of species (Walker *et al.*, 2012; Andrews *et al.*, 2015, 2019; Shuert *et al.*, 2015; Chivers *et al.*, 2016; Horning *et al.*, 2017a; Lear *et al.*, 2018). Even when not found to impact survival, physiological costs associated with telemetry devices likely exist with respect to increased drag associated with the disruption of laminar flow across the body (Andrews *et al.*, 2015; Andrews and Enstipp, 2016; Kyte *et al.*, 2019). Effect sizes associated with the ‘bolt-on’ tags here were very small with respect to their impact on swimming behaviour, and we failed to find a significant relationship between ‘bolt-on’ tags and recovery time. While our overall sample size of individuals is large compared to other studies, it is unlikely that our models had sufficient power to detect any major behavioural differences as a result of narwhal carrying a ‘bolt-on’ satellite tag over longer time scales.

Alongside the attachment of a ‘bolt-on’ satellite tag, there was also evidence to suggest that individual sex had an influence on all accelerometry-derived behaviours in the first 72 hours after release. Models including individual sex and the presence of a ‘bolt-on’ satellite tag were apparently equal in their explanatory power for swimming behaviour (ΔAICc < 2; Table 2). Females were found to have both lower activity levels and tail-beat frequencies, but this did not appear to have a significant effect on recovery time. While age class was ultimately removed, individuals with satellite tags were larger, older individuals who may simply exhibit slower tailbeat frequencies. Larger whales can be assumed to have larger peduncle muscles in which to create a greater output of momentum for each stroke (Goldbogen *et al.*, 2006). This has been demonstrated in experimental studies of fish species where increased body length scales as a power equation for both propulsive force, as well as a decrease in the frequency of strokes (Bainbridge, 1958; Ware, 1978; Webb *et al.*, 1984; Ohlberger *et al.*, 2007). Increased body size has been shown to result in a logarithmic decrease in the cost of transport in aquatic animals, defined as the energy required to move a unit of body mass a unit distance (Schmidt-Nielsen, 1972; Fish, 1994). Mammalian studies have used captive surrogates to scale up stroke-by-stroke energy costs to other species and have shown similar results (Williams *et al.*, 2004, 2017b). While it was not possible to age these individuals beyond coarse size classes, it is likely that this change in both activity levels and swimming behaviour is a result of differences in body size and development (Noren *et al.*, 2006), but requires further investigation.

Long-term attachments of telemetry devices, through invasive procedures such as ‘bolt-on’ configurations remain an important tool for understanding aspects of marine animal migrations, habitat use, site fidelity, and year-round behaviour and can greatly contribute to the conservation efforts of many species (Hussey *et al.*, 2015; Hazen *et al.*, 2017; Andrews *et al.*, 2019). Long-term deployments often require active capture and handling in order to attach the devices. Consideration should therefore be given to determine if such invasive techniques are necessary in order to achieve the goals of the proposed work (Andrews *et al.*, 2019); planning requires thorough and transparent discussion of questions and tagging approaches as well as potential outcomes of the tagging mode used with all involved stakeholders. Under certain scenarios, short-term deployments via suction cups or remote tagging can address study questions effectively (e.g. Goldbogen *et al.*, 2013), whereby the impact of tagging on behaviour is minimal and therefore data loss related to non-normal behaviour is minimal. Addressing questions such as migration timing and extent, however, will require more invasive ‘bolt-on’ tagging procedures unless alternative long-term attachment methods are developed (Heide-Jørgensen *et al.*, 2017). Opportunities to advance long-term attachment methods will require broader discussion among the scientific community and tag manufacturers that will likely lead to smaller design and more battery efficient tags (as discussed in Andrews *et al.*, 2019; Horning *et al.*, 2019). This will also require that tag manufacturers are willing to invest in research and development of new tags and attachment methods. For the Arctic whales, including traditional Inuit Qaujimajatuqangit could lead to the development and design of novel dart heads for long-term deployments via remote tag attachment given their wealth of experience with harpoon use (Noongwook *et al.*, 2007; Idrobo and Berkes, 2012; Johnson *et al.*, 2015; Pearce *et al.*, 2015; Pedersen *et al.* 2020).

Future climate scenarios (Williams *et al.*, 2011) and an increase in the incidence of disease in the Arctic (VanWormer *et al.*, 2019) further highlights the importance of considering the implications of capture and tagging practices, the sample sizes required to answer the question at hand (Sequeira *et al.*, 2019), and whether alternative tagging methods without the need for capture may be more appropriate (Best *et al.*, 2015; Seyboth *et al.*, 2017). If, for example, ‘bolt-on’ tag configurations are required to investigate stock structure in narwhal in the Arctic, researchers should consider equipping a small sample size of animals with accelerometer packages in conjunction with satellite tags and undertaking initial field investigations of data following the above approaches to determine impact (Wilson and McMahon, 2006). Evidence-based effects of capture, handling and tagging generated while in the field would then provide confidence in the approach, while considering the welfare of the animals related to uncertainties such as impacts of climate change and remains a key recommendation of best practice guidelines (Wilson and McMahon, 2006; Horning *et al.*, 2017b, 2019; Andrews *et al.*, 2019). Supporting the work of previous studies, we recommend that every effort should be taken to minimize handling time if active capture and ‘bolt-on’ tag placement is required. We also advocate further investigation on how to mitigate the potential effects of ‘bolt-on’ tags through modified designs, approaches and rigorous experimental design in the field in order to strive for continued refinement in biologging applications (Hawkins, 2004; Casper 2009), especially for sensitive species such as narwhal (Laidre *et al.*, 2008).

## Funding

This work was supported by the NSERC Northern supplement and NSERC Discovery funds to N.E.H. C.R.S was supported by Mitacs through the Mitacs Accelerate program (www.mitacs.ca) in partnership with the World Wildlife Fund. M.A.M. thanks the Canadian Research Chairs program. Funding for fieldwork was provided by Fisheries and Oceans Canada, the Nunavut Wildlife Research Trust, and the World Wildlife Fund.

## Supplementary Material

C_Shuertetal_SUPP_Narwhal_post_release_behaviour_final_coaa128
