# Supplementary material for: Assessing the post-release effects of capture, handling and placement of satellite telemetry devices on narwhal (Monodon monoceros) movement behaviour
Source: Conserv Physiol. 2021 Jan 7;9(1):coaa128. doi: 10.1093/conphys/coaa128 (PMC7905160; doi:10.1093/conphys/coaa128)
Supplement: C_Shuertetal_SUPP_Narwhal_post_release_behaviour_final_coaa128 [file c_shuertetal_supp_narwhal_post_release_behaviour_final_coaa128.docx]

**SUPPLEMENTARY MATERIALS**

**Title**: Assessing the post-release effects of capture and handling on narwhal behaviour following placement of satellite telemetry devices.

Running Title: Narwhal post-release behaviour

**Authors:** Courtney R. Shuert^1,2,*^, Marianne Marcoux^2^, Nigel E. Hussey^1^, Cortney A. Watt^2,3^, Marie Auger-Méthé^4,5^

^1^ Department of Integrative Biology, University of Windsor, Windsor, ON, Canada N9B 3P4

^2^ Arctic Aquatic Research Division, Fisheries and Oceans Canada, Winnipeg, MB, Canada R3T 2N6

^3^ Department of Biological Sciences, University of Manitoba, Winnipeg, MB, Canada R3T 2N2

^4^ Department of Statistics, University of British Columbia, Vancouver, BC, Canada V6T 1Z4

^5^ Institute for the Oceans & Fisheries, University of British Columbia, Vancouver, BC, Canada V6T 1Z4

^*^ corresponding author: [cshuert@gmail.com](mailto:cshuert@gmail.com)

**Lay Summary:** Narwhals, iconic Arctic cetaceans, were monitored after routine capture and tagging. Using accelerometer-derived behaviour, most individuals appeared to recover within hours post-release. Some evidence suggests that handling time, sex, and the presence of bolt-on satellite tags may had an effect on changes in behaviour, highlighting the sensitivity of this species.

**Table S1:** Model output for top models describing hourly post-release activity, energy expenditure, and swimming behaviour using generalized additive models fit via thin-plate regression splines with shrinkage. Best models for post-release behaviour were best described by the parametric effects of handling time, *t_cap_*, (short, < 30 min; medium, 30-40 min; long, > 40 min) individual sex and the presence of a ‘bolt-on’ satellite tag (Sat.Tag). Smoothing-splines describing behaviour as a function of time since release (*t_rel_*) in top models were found to vary by coefficients of sex and handling time.

| **Activity (norm of jerk, g/s)** | | | |  |  |  |  |  |  |  |  |  |  |
| --- | --- | --- | --- | --- | --- | --- | --- | --- | --- | --- | --- | --- | --- |
| Param. Terms | Est. | t | *p* |  |  |  |  |  |  |  |  |  |  |
| Intercept | -0.22 (0.05) | -3.78 | < 0.001 |  |  |  |  |  |  |  |  |  |  |
| *t_cap_*:Medium | -0.24 (0.07) | -3.17 | < 0.001 |  |  |  |  |  |  |  |  |  |  |
| *t_cap_*:Long | 0.26 (0.11) | 2.30 | 0.02 |  |  |  |  |  |  |  |  |  |  |
| Smooth Terms | edf | F | *p* |  |  |  |  |  |  |  |  |  |  |
| s(*t_rel_*):Male | 7.26 | 8.91 | < 0.001 |  |  |  |  |  |  |  |  |  |  |
| s(*t_rel_*):Female | 2.57 | 2.76 | < 0.001 |  |  |  |  |  |  |  |  |  |  |
|  |  |  |  |  |  |  |  |  |  |  |  |  |  |
| **Energy Expenditure (sVeDBA, g)** | | |  |  |  |  |  |  |  |  |  |  |  |
| Param. Terms | Est. | t | *p* |  | Smooth Terms | edf | F | *p* |  |  |  |  |  |
| Intercept | -0.01 (0.003) | -4.19 | < 0.001 |  | s(*t_rel_*):Male | 0.0006 | 0 | 0.92 |  |  |  |  |  |
| t*_cap_*:Medium | -0.01 (0.004) | -2.73 | 0.006 |  | s(*t_rel_*):Female | 2.39 | 3.94 | < 0.001 |  |  |  |  |  |
| *t_cap_*:Long | -0.004 (0.006) | -0.62 | 0.53 |  |  |  |  |  |  |  |  |  |  |
| Smooth Terms | edf | F | *p* |  |  |  |  |  |  |  |  |  |  |
| s(*t_rel_*):Male | 0 | 0 | 0.89 |  |  |  |  |  |  |  |  |  |  |
| s(*t_rel_*):Female | 2.35 | 4.3 | < 0.001 |  |  |  |  |  |  |  |  |  |  |
|  |  |  |  |  |  |  |  |  |  |  |  |  |  |
| **Swimming Behaviour (TBFreq, Hz)** | | | |  |  |  |  |  |  |  |  |  |  |
| Smooth Terms | edf | F | *p* |  | Param. Terms | Est. | t | *p* |  | Param. Terms | Est. | t | *p* |
| s(*t_rel_*):Short | 2.02 | 0.95 | 0.008 |  | Intercept | 0.03 (0.01) | 2.77 | 0.005 |  | Intercept | 0.02 (0.01) | 2.08 | 0.03 |
| s(*t_rel_*):Medium | 0.95 | 1.23 | < 0.001 |  | Sex(F) | -0.04 (0.01) | -3.19 | 0.001 |  | Sat.Tag | -0.03 (0.01) | -2.55 | 0.01 |
| s(*t_rel_*):Long | 5.91 | 11.2 | < 0.001 |  | Smooth Terms | edf | F | *p* |  | Smooth Terms | edf | F | *p* |
|  |  |  |  |  | s(*t_rel_*):Short | 2.1 | 1.06 | 0.005 |  | s(*t_rel_*):Short | 2.1 | 1.35 | 0.001 |
|  |  |  |  |  | s(*t_rel_*):Medium | 0.92 | 0.91 | 0.002 |  | s(*t_rel_*):Medium | 0.92 | 0.94 | 0.002 |
|  |  |  |  |  | s(trel):Long | 5.6 | 6.37 | < 0.001 |  | s(trel):Long | 6 | 12.11 | < 0.001 |

**Table S2:** Model selection results describing covariate effects on time (hrs) to recovery in narwhals following routine capture, handling, and tagging operations using generalized linear models with a Poisson distribution. Covariates included animal sex (female as positive condition), the presence of a ‘bolt-on’ satellite tag (Sat.Tag), and the handling time (*t_cap_*) binned into short (< 30 min), medium (30-40 min), and long durations (> 40 min). None of the covariates measured appear to have an effect on the time to recovery.

| Model | Effect Size | df | Residual Deviance | ΔAICc | AICc |
| --- | --- | --- | --- | --- | --- |
| Null | - | 15 | 104.72 | 0 | 163.3 |
| Sex | 0.21 (0.18) | 14 | 103.35 | 1.5 | 164.8 |
| Sat.Tag | -0.10 (0.21) | 14 | 104.46 | 2.6 | 165.9 |
| *t_cap_* | S:M 0.32 (0.19)  S:L 0.10 (0.30) | 13 | 101.62 | 2.9 | 166.2 |
